# Using Screen Printing Technology to Fabricate Flexible Sodium Ion Sensors

**DOI:** 10.3390/s25123650

**Published:** 2025-06-11

**Authors:** Fang-Hsing Wang, Shang-Wei Huang, Cheng-Fu Yang, Kao-Wei Min

**Affiliations:** 1 Graduate Institute of Optoelectronic Engineering, National Chung Hsing University, Taichung 402, Taiwan; 2 Department of Chemical and Materials Engineering, National University of Kaohsiung, Kaohsiung 811, Taiwan; 3 Department of Aeronautical Engineering, Chaoyang University of Technology, Taichung 413, Taiwan; 4 Department of Electrical Engineering, Lunghwa University of Science and Technology, Taoyuan 333, Taiwan

**Keywords:** Na^+^ ion sensing devices, working electrode, graphene oxide (GO), multi-walled carbon nanotubes (MWCNTs), selectivity, response time

## Abstract

**Highlights:**

**What are the main findings?**

- Incorporating graphene oxide (GO) or multi-walled carbon nanotubes (MWCNTs) into the sensing electrode significantly influenced the Na^+^ ion sensing performance.
- Sensors with MWCNTs exhibited improved sensing characteristics, including faster response times and enhanced selectivity for Na^+^ ions.

**What are the implications of the main findings?**

- The improved performance of Na^+^ ion sensors with MWCNTs suggests that carbon nanomaterials, particularly MWCNTs, are promising additives for enhancing ion-selective sensor functionality. This advancement could contribute to the development of more sensitive and selective electrochemical sensors for biomedical or environmental monitoring applications.

**Abstract:**

This study focused on the development of Na^+^ ion sensing devices on a flexible substrate and investigated the impact of various additive materials on its sensing performance. For the Na^+^ ion sensing aspect, the film on the carbon working electrode used tert-butyl calix[4]arene tetraethyl acetate as the ion carrier. The main component of the film was polyvinyl chloride (PVC), with a plasticizer added to enhance its flexibility, ensuring better adaptation to the flexible substrate. In this base formulation, graphene oxide (GO) or multi-walled carbon nanotubes (MWCNTs) were incorporated into the sensing electrode to explore their effects on Na^+^ ion sensing capabilities. The results demonstrated that adding MWCNTs significantly improved the sensor’s sensitivity to Na^+^ ions. In addition, the study used the response slope to Na^+^ ions as a comparative reference for selectivity by calculating the ratio of the Na^+^ ion response slope to the response slopes of other ions (such as K^+^ and Ca^2+^). The findings showed that the sensors with MWCNTs exhibited better selectivity than the others with GO, and therefore, further analysis was performed on the response time of the sensors with MWCNTs. The results indicated that incorporating MWCNTs reduced the sensors’ response time and enhanced their overall sensitivity. However, excessive addition of MWCNTs would lead to a decrease in the selectivity of the fabricated sensors. This suggests that while MWCNTs offer promising improvements in performance, their concentration must be carefully optimized to maintain the sensors’ selectivity.

## 1. Introduction

Traditional medicine typically uses invasive methods to obtain blood or bodily fluids for analysis to assess the health status of individuals. However, these methods require expensive equipment and complex testing procedures, making it difficult to achieve real-time and continuous monitoring. Future research will focus on developing non-invasive, wearable continuous sensing technologies. Currently, most wearable sensors on the market are limited to tracking physical activity and simple health data, such as steps or heart rate, and cannot provide insights into the user’s health at the molecular level. In contrast, measuring human sweat through non-invasive methods can offer this information, as sweat contains rich physiological and metabolic data. The Na^+^ ion concentration in sweat can be used to assess an athlete’s electrolyte balance and hydration status, providing insights into fluid loss and needs [1]. Sweat analysis has already been applied in disease diagnosis and evaluating physical condition during exercise. However, current methods typically require separate collection and analysis of samples, preventing real-time monitoring and necessitating expensive equipment. Although some technologies have been developed to analyze sweat components [2], research on biological fluids remains limited, making the practical implementation of sweat-sensing systems a significant challenge.

Over the past 30 years, ion-selective electrodes (ISEs) using ion carriers have developed into a mature and widely used analytical technique [3]. For example, a 1980 survey by the American Academy of Pathology revealed that only 22% of laboratories at that time performed Na^+^ or potassium (K^+^) potential measurements [4]. Due to the use of electrode systems made with various polymer membranes to monitor other biologically relevant ions, over 1 billion ISE measurements are performed annually in clinical laboratories worldwide [5]. ISEs consist of a reference electrode and a working electrode. The reference electrode must exhibit high stability and be reusable, maintaining a constant potential despite changes in ion concentration. The working electrode, which includes a sensing membrane and a conductive layer, should show a regular change in membrane potential in response to variations in the target ion concentration and remain unaffected by changes in the concentration of interfering ions. When both electrodes are immersed in a solution to be tested, the potential difference between them is used to measure ion concentration. Therefore, the stability of the electrode potential is crucial for accurate ion concentration measurements [6]. In the past, many methods have investigated wearable Na^+^ ion sensors. Lim et al. explored the development of a flexible and hydrophobic composite material, combining carbon black and a soft elastomer, which functions as an efficient ion-to-electron transducer. This innovative sensor design offers several advantages, including cost-effectiveness, simplicity in fabrication, and long-term stability, making it a promising option for a wide range of applications [7].

Pfeffer et al. proposed an advanced sensor stack, which they optimized using finite element method (FEM) simulations to enhance its performance and functionality. Following the optimization, they fabricated the new sensor and conducted measurements using electrochemical impedance spectroscopy (EIS) to evaluate its efficacy and performance in real-world conditions [8]. The use of FEM simulations allowed for precise design adjustments, ensuring that the sensor’s sensitivity and accuracy were maximized. Ghoorchian et al. introduced a wearable potentiometric ion sensor designed for real-time monitoring of Na^+^ ions in human sweat samples. The sensor utilized Na_0.44_MnO_2_ as the sensing material, chosen for its excellent Na^+^ incorporation capacity, which makes it highly suitable for wearable electrochemical sensors. Na_0.44_MnO_2_’s ability to efficiently interact with Na^+^ ions and provide high sensitivity and stability is crucial for continuous and accurate monitoring in dynamic biological environments [9]. Large-area graphene has emerged as an ideal material for high-resolution ion-sensitive field-effect transistors (ISFETs), offering exceptional performance while also facilitating easier fabrication compared to conventional semiconductor materials. Its unique properties, including high electrical conductivity, flexibility, and ease of integration into large-scale devices, make it highly suitable for advanced sensor applications. Building on these advantages, Jalal et al. advanced the ISFET technology by developing a sensor array based on graphene, which enabled improved sensitivity and resolution [10].

This study is primarily focused on the development of flexible Na^+^ ion sensors, laying the groundwork for the future creation of wearable Na^+^ ion sensors. On the working electrode, we applied a composite material as the selective membrane. The main component of the membrane was polyvinyl chloride (PVC), combined with plasticizers, sodium (Na^+^) ion carriers, anion exchangers, and other additives [11,12]. The first key innovation of this research lies in the selection of PVC as the base material for the membrane, which was further enhanced by the addition of plasticizers to increase the flexibility of the film. This modification allows the membrane to better conform to flexible substrates, making it ideal for wearable applications. Tetrahydrofuran (THF) was employed as the solvent to dissolve these materials. PVC, as a polymer, does not form a single, uniform mass but rather exists in a distributed form, with the molecular weight of the polymer playing a crucial role in determining the mechanical strength of the membrane. For this study, a molecular weight of approximately 47,000 was chosen, which is considered moderately high [11,12]. This selection strikes an optimal balance, ensuring that the membrane maintains satisfactory performance without introducing significant challenges during the manufacturing process. The second major innovation of this study involves the incorporation of graphene oxide (GO) or multi-walled carbon nanotubes (MWCNTs) into the existing formulation. The addition of MWCNTs significantly reduces the response time of the sensor while simultaneously enhancing its sensitivity. The impact of MWCNTs on the overall Na^+^ ion sensing efficiency will be thoroughly examined and analyzed in detail throughout this study, highlighting the substantial improvements in performance they bring to the sensor.

## 2. Materials and Methods

**Figure 1:**
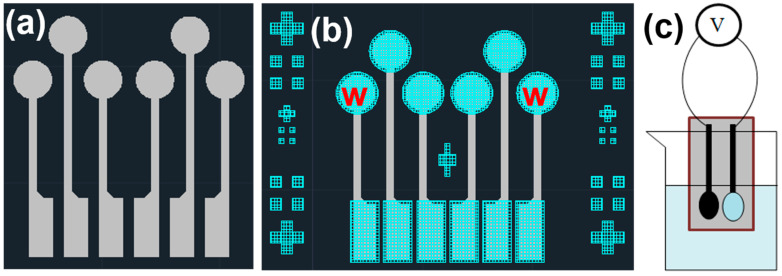
Layout diagrams of (**a**) the electrode platform’s conductive layer (gray) and (**b**) the electrode platform’s second layer (blue), and (**c**) a schematic diagram of the open-circuit voltage measurement.

In this study, screen printing was used to fabricate electrodes on PET, and the design was created using AutoCAD (version 24.1) software. Figure 1a shows the first conductive layer used, which was made of silver. The upper circular sensing area had a diameter of 3.2 mm, and the width of the central lines was 0.6 mm, with lengths of 12 mm and 8 mm, respectively. The lower rectangular measurement area was 2 mm × 5 mm, and the sheet resistance of this layer, as measured, was 160 mΩ per square. The second layer material was carbon, which served to protect the underlying silver layer while maintaining electrical conductivity, and the layout diagram is shown in Figure 1b. The upper circular sensing area had a diameter of 3.8 mm, and the lower rectangular measurement area had dimensions of 2.6 mm × 5.6 mm. The sheet resistance of this layer, as measured, was 98 Ω per square. In this study, the ion carrier used was 4-tert-butylcalix[4]arene-tetraacetic acid tetraethyl ester. The calixarene component is a macrocyclic molecule synthesized from p-tert-butylphenol and an aldehyde under basic conditions. It is named “calixarene” due to its cup-like structure, which resembles the shape of a cup. The solution used for creating the working electrode’s thin film consisted of 1320 mg (33 wt%) PVC, 2620 mg (65.5 wt%) DOS, 40 mg (1 wt%) Na^+^ ion carrier, and 20 mg (0.5 wt%) ion exchanger. These chemicals were sequentially added to 50 mL of THF. Before adding each material, the mixture was stirred for five minutes using a magnetic stirrer to ensure complete dissolution of the components into the solvent, forming a transparent and clear solution. As a variable, 0/4/8 mg of GO or MWCNTs were added, creating a total of six conditions to explore their effect on the sensing characteristics of the fabricated flexible Na^+^ ion sensors.

This formulation and its usage were based on the work of Heng and Lee [13], who tested it and found it to have the best response slope and ion selectivity. The coating method followed the approach of Roy et al. [14]. After preparing the aforementioned materials, a concentrated thin-film solution with a concentration of 0.08 g/mL was obtained (concentrated thin-film solution). A small amount of this solution was then taken and diluted with THF in a 9:1 ratio, increasing the volume by ten times (diluted thin-film solution), which reduced its concentration to 0.008 g/mL. The diluted thin-film solution was then applied onto the working electrode. A volume of 2.5 μL was deposited each time, ensuring that the solution completely covered the carbon electrode at the bottom. The solution was left to dry for ten minutes, forming a thin film. This step was repeated five times with the diluted thin-film solution. Afterward, the concentrated thin-film solution was used, applying 2.5 μL per drop, with a ten-minute waiting period for drying. This coating method aims to enhance the adhesion between the film and the underlying carbon electrode to prevent the film from peeling off after multiple uses. Next, the concentrated thin-film solution was applied twice more. The final application of the concentrated solution is essential because the diluted thin film has more and larger pores.

During NaCl solution sensing experiments, these pores may trap a significant amount of ions from the previous test solution, making it difficult for the new test solution to replace the old one. This would limit the ion-selective electrode’s sensitivity to NaCl. Therefore, the concentrated thin-film solution was applied in the final step to serve as the sensing layer between the working electrode and the solution. After this step, the working electrode used in this experiment was complete. Next, the open-circuit voltage was measured using a Keithley 2450 (Keithley Instruments, Cleveland, OH, USA). The related analyses included the voltage response to the concentrations of different ions (Na^+^, K^+^, and Ca^2+^), response time, and the device’s selectivity to other ions. For different ion concentrations, standard solutions purchased and deionized water were used for preparation. For testing at different pH levels, ammonia and nitric acid were mixed with deionized water to avoid the unknown concentration of Na^+^ ions that might be present in purchased pH buffer solutions. In observing the measurement limits, conditions where the voltage fluctuation did not exceed 10 mV were considered valid data. Figure 1c shows a schematic of the open-circuit voltage measurement. The working electrode and reference electrode were placed in the same solution, and the instrument was used to measure the potential difference between the two electrodes, which indirectly indicated the Na^+^ ion concentration. The Na^+^ ion sensor consisted of a working electrode and a reference electrode (indicated by the red “w” in Figure 1b). The working electrode was formed by applying a solution we prepared onto the carbon electrode.

## 3. Results and Discussion

**Figure 2:**
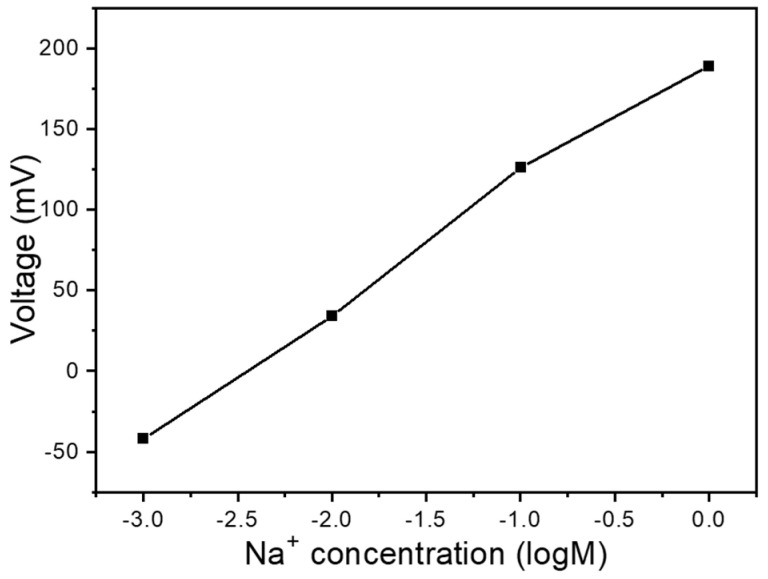
Na^+^ ion reaction curve using C as the electrode.

Our investigation of reference electrodes involved comparing the carbon electrode from the original substrate. This setup utilized the same working electrode position, as Figure 1b,c shows, under conditions without any additives such as GO or MWCNTs. Figure 2 presents a slope comparison between these two reference electrodes across a concentration range from 10^–3^ M to 1 M. When the carbon electrode served as the reference electrode, we observed voltage changes from −42 mV to 189 mV, with a response sensitivity of 77.0 mV/logM and a linearity of 0.982. These results clearly demonstrate that employing the carbon electrode as the reference enhances response sensitivity while providing superior linearity. This phenomenon likely occurs because the carbon electrode exhibits a minor response to Na^+^ ions, with this response oriented in the opposite direction to that of the working electrode. This opposing response mechanism enables measurements to surpass theoretical predictions, thereby enhancing overall sensitivity. The implications of these findings extend beyond mere performance metrics. The enhanced sensitivity and linearity offered by carbon reference electrodes could prove particularly valuable in applications requiring the detection of low Na^+^ ion concentrations.

**Figure 3:**
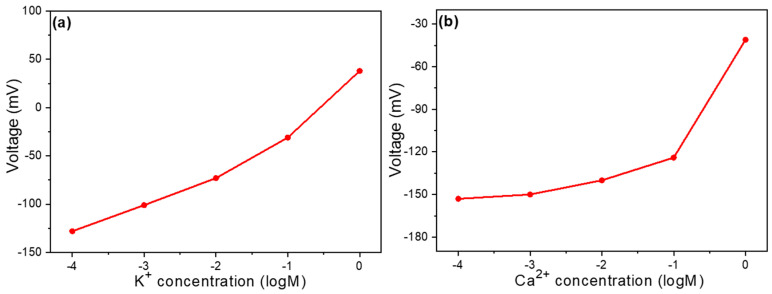
Voltage vs. concentration curve of a Na^+^ ion sensor for (**a**) K^+^ ions and (**b**) Ca^2+^ ions.

Ion selectivity is a crucial characteristic for precision. In this regard, we first tested the differences between reference electrodes. As mentioned earlier, using carbon electrodes enhances the response to Na^+^ ions. Here, we aim to determine whether the use of carbon electrodes compromises ion selectivity. Figure 3a shows the voltage measurements for different K^+^ ion concentrations with the carbon reference electrode, corresponding to the same working electrode (0 doping). Ion selectivity is considered good when the device exhibits a clear response only to the target ion. In this plot, the concentration of K^+^ ions is varied, but since our target ion is Na^+^, we would prefer the slope to be as small as possible. A smaller slope would indicate better selectivity. From the graph, we can observe that the device using a carbon reference electrode has a relatively small slope. Figure 3a presents the voltage response across a concentration range from 10^–4^ M to 1 M. We observed that the voltage shifted from −128 mV to 38 mV, with a response sensitivity of 41.5 mV/logM. For selectivity testing, we also examined calcium (Ca^2+^) ions. Figure 3b shows the selectivity measurements for Ca^2+^ ions under the same experimental conditions as those in Figure 3a. The voltage change is clearly divided into two segments. As the concentration changed from 10^–4^ M to 10^–1^ M, the voltage shifted from −153 mV to −124 mV, with a response sensitivity of 9.67 mV/logM. As the concentration increased from 10^–1^ M to 1 M, the voltage dramatically shifted from −124 mV to −41 mV. This demonstrates that when using a carbon-based reference electrode, the device exhibits a small slope for Ca^2+^ ion concentrations below 10^–1^ M, indicating good selectivity in this range. Therefore, this device is considered to have good ion selectivity when the Ca^2+^ ion concentration is lower than 10^–1^ M.

This finding has significant implications for sensor design and optimization. The superior performance of carbon electrodes in maintaining selectivity for Na^+^ ions while minimizing interference from K^+^ and Ca^2+^ ions suggests several underlying mechanisms. The electrochemical properties of carbon likely create a more stable interface with the analyte solution, resulting in more consistent baseline measurements against which Na^+^ ion concentrations can be accurately determined. From a practical perspective, carbon electrodes offer additional advantages beyond selectivity. Their chemical stability, mechanical robustness, and cost-effectiveness make them attractive candidates for widespread adoption in Na^+^-sensing applications. Furthermore, carbon electrodes are environmentally friendly compared to silver-based alternatives, aligning with sustainability goals in modern sensor development. Future work should investigate whether this enhanced selectivity extends to other potentially interfering ions in complex biological samples. Additionally, exploring different carbon materials (e.g., graphene, carbon nanotubes, or activated carbon) could further optimize selectivity and sensitivity parameters for specific applications such as clinical diagnostics or environmental monitoring.

**Figure 4:**
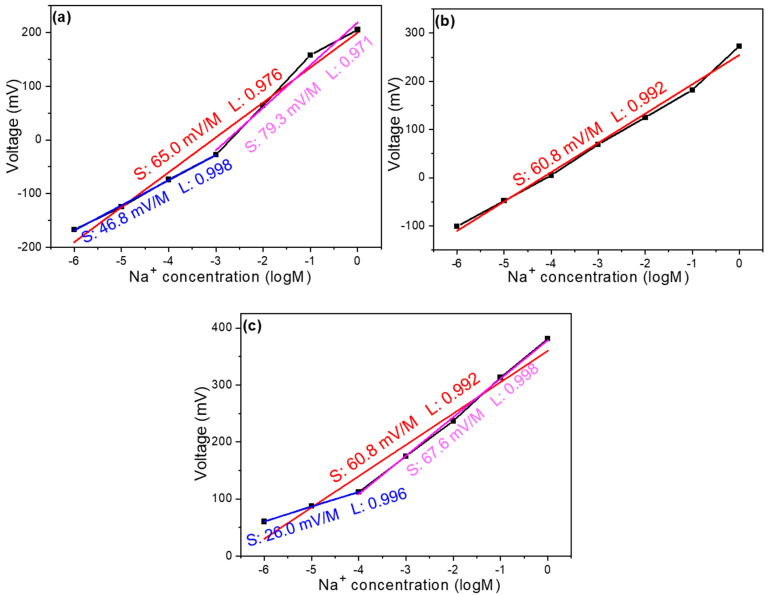
Concentration response curve of a Na^+^ ion sensor (**a**) without the addition of GO, with the addition of (**b**) 4 mg GO and (**c**) 8 mg GO.

After confirming the use of carbon as the reference electrode, we proceeded to test how different doping conditions affect selectivity. Figure 4a shows the concentration–voltage response curves of two identical sensing devices fabricated under the same processing conditions, both using pure thin films without any additives and the same reference electrode. From these concentration–voltage curves, the slope represents the device’s response sensitivity—a larger slope indicates better response performance. The detection limit ranges from 1 M to 10^−6^ M, with an overall slope of 65.0 mV/dec, meaning that for every 10-fold change in concentration, the voltage increases or decreases by 65 mV. The overall linearity is 0.976. Upon closer examination, the response can be divided into two linear regions: in the concentration range of 1 M to 10^–3^ M, the response is 79.3 mV/dec with a linearity of 0.971, while in the concentration range of 10^–3^ M to 10^–6^ M, the response is 46.8 mV/dec with an excellent linearity of 0.998. Figure 4b,c represents the relationship between open-circuit voltage and concentration for thin films with 4 mg and 8 mg of GO added to the film solution, respectively. After adding 4 mg of GO, the linear range of the sensor increased to span from 1 M to 10^–6^ M.

The overall slope became more consistent throughout this range, although it decreased slightly to 60.79 mV/dec. This suggests that while the addition of GO improves the sensor’s linear operating range, it slightly reduces its sensitivity. For the device with 8 mg of GO, the linear range starting from 1 M was slightly lower than that of the 4 mg sample, but still notably larger than the device without GO. The overall slope decreased further to 55 mV/dec with a linearity of 0.962. When examined in detail, the response can be divided into two regions: between 1 M and 10^–4^ M, the slope is 67.6 mV/dec with an excellent linearity of 0.998, while between 10^–4^ M and 10^–6^ M, the slope decreases significantly to 26 mV/dec, maintaining a linearity of 0.998. These results demonstrate that the addition of GO can effectively expand the linear detection range of the sensor, which is beneficial for broadening its application scope. However, this comes at the cost of reduced sensitivity (lower slope values), with sensitivity decreasing more noticeably as the amount of GO increases.

The addition of GO increases the linear range but decreases the response primarily because the surface of GO contains a large number of oxygen-containing functional groups (such as carboxyl, hydroxyl, and epoxy groups). These functional groups interact with ions in the solution, allowing the sensing reaction to remain linear over a wider concentration range. However, they may also partially obstruct ion access to the electrode surface, slowing down the charge transfer process. Additionally, GO has relatively poor electrical conductivity. Although it increases the surface area, its sp^3^ hybridized structure reduces electron transport efficiency. This lower conductivity may reduce the sensitivity of potential changes to concentration variations. Moreover, charge shielding layers may form between the GO sheets, weakening the effect of external ion concentration changes on the electrode. The more GO is added, the more pronounced this shielding effect becomes, leading to a more significant decrease in the slope. Notably, although the overall linear detection range is expanded with GO, the sensor with 8 mg GO exhibits a narrower linear range in the low-concentration region compared to the sensor without GO, as also observed in Figure 4. This implies that excess GO may adversely affect ion accessibility and signal response in the dilute regime. Furthermore, GO/s porous structure may alter the ion diffusion characteristics on the film surface, maintaining consistent ion diffusion over a wider concentration range (improving the linear range). However, this may also increase diffusion resistance, lowering the reaction speed and sensitivity. The greater the amount of GO added, the more pronounced these effects become. For instance, the slope at 8 mg is lower than at 4 mg, indicating the presence of an optimal addition amount. Exceeding this amount leads to a decline in performance. This trade-off between linear range and sensitivity—especially at lower concentrations—represents an important design consideration for optimizing sensor performance for specific applications.

**Figure 5:**
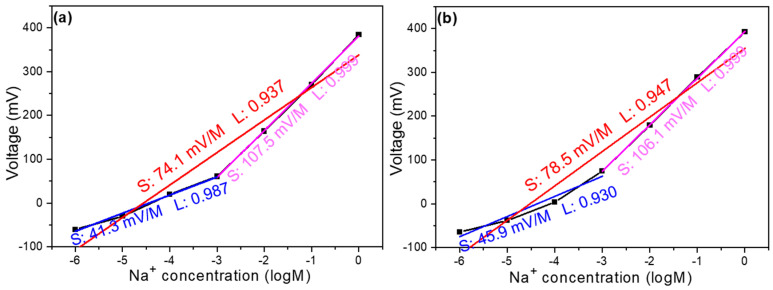
Concentration response curve of a Na^+^ ion sensor with the addition of (**a**) 4 mg MWCNTs and (**b**) 8 mg MWCNTs.

Figure 5a,b illustrates the relationship between open circuit voltage and concentration in thin film solutions with the addition of 4 mg and 8 mg of MWCNTs, respectively. The observed trends share similarities with the characteristics of samples without any additives, maintaining the same detection limit range of 1 M to 10^–6^ M. Upon closer examination, the response can be divided into two distinct linear regions. When 4 mg of MWCNTs was incorporated, the overall slope reached 74.14 mV/dec with a linearity of 0.937. Breaking this down further reveals that the high-concentration region (1 M to 10^–3^ M) exhibited a slope of 107.5 mV/dec with excellent linearity (0.999), while the low-concentration region (10^–3^ M to 10^–6^ M) showed a slope of 41.3 mV/dec with a linearity of 0.987. Similarly, with 8 mg of MWCNTs, the overall slope increased to 79.1 mV/dec with a linearity of 0.946. The high-concentration region maintained a comparable slope of 107.4 mV/dec with a linearity of 0.999, while the low-concentration region demonstrated a slope of 45.5 mV/dec with a linearity of 0.929.

The addition of MWCNTs has notably enhanced both the overall and regional slopes compared to the pristine samples. This improvement is particularly advantageous for our sensing applications, as steeper slopes generally indicate higher sensitivity in electrochemical sensors. The enhanced response suggests that MWCNTs are effectively improving the electrical conductivity and electrochemical properties of the thin film, potentially creating more efficient electron transfer pathways that amplify the voltage response to concentration changes. The addition of MWCNTs leads to an increase in the slope, primarily due to the excellent electrical conductivity of the nanotubes and their ability to provide a larger surface area, which enhances the electronic transport properties of the film and improves the contact area between ions and the electrode interface. Additionally, MWCNTs possess catalytic properties, which can alter the microstructure of the film/solution interface, accelerating the charge transfer process at the interface and making ion adsorption/desorption more sensitive.

To clarify the performance difference between GO and MWCNTs, we provide the following mechanistic explanation based on the experimental results and established material properties:

1. Electrical conductivity: MWCNTs possess superior electrical conductivity compared to GO (graphene oxide), primarily due to their highly conjugated sp^2^ carbon structure with minimal oxygen-containing functional groups. This inherent high conductivity facilitates more efficient electron transport within the sensing membrane, leading to enhanced electrochemical transduction. This is reflected in our results: devices containing MWCNTs exhibited higher sensitivity, as indicated by steeper slopes (e.g., 107.5 mV/dec and 107.4 mV/dec for high-concentration regions), compared to GO-containing devices, which showed lower slopes (67.6 mV/dec and 26 mV/dec in corresponding regions). The enhanced conductivity of MWCNTs accelerates charge transfer between the analyte and the sensing layer, thereby improving sensitivity.

2. Interfacial interaction and surface area: MWCNTs also offer excellent interfacial contact with the matrix due to their high aspect ratio and one-dimensional nanostructure. This morphology promotes the formation of effective percolation networks at relatively low loadings, enhancing signal propagation pathways. Additionally, MWCNTs interact with the surrounding matrix and analytes through π-π stacking and van der Waals forces, which contributes to better charge carrier mobility. In contrast, GO contains a high density of oxygenated functional groups (e.g., -OH, -COOH, epoxy), which disrupt its π-conjugation and lower its intrinsic conductivity. However, these groups enhance hydrophilicity and dispersion within the matrix, which helps increase analyte accessibility and homogeneity of the membrane. This improved dispersion accounts for the expanded linear detection range observed with GO (e.g., linear region extended down to 10^−6^ M), as it facilitates uniform interaction between the sensing membrane and target molecules.

3. Trade-off between sensitivity and linear range: The results suggest a clear trade-off: MWCNTs enhance sensitivity (higher slope) due to their superior conductivity and interfacial charge transport properties, whereas GO improves the linear detection range due to its better dispersion and analyte accessibility. For example, the 8 mg MWCNT device exhibited a high slope of 107.4 mV/dec in the high-concentration region, whereas the 8 mg GO device showed a broader linear range extending from 1 M to 10^−6^ M, but with a much lower slope (26 mV/dec in the low-concentration region).

MWCNTs outperform GO in terms of sensitivity due to their better electrical conductivity and interfacial charge transport efficiency, whereas GO contributes more to extending the linear detection range through enhanced dispersion and analyte interaction. These mechanistic insights provide a clearer understanding of the complementary roles played by MWCNTs and GO in sensor performance.

**Figure 6:**
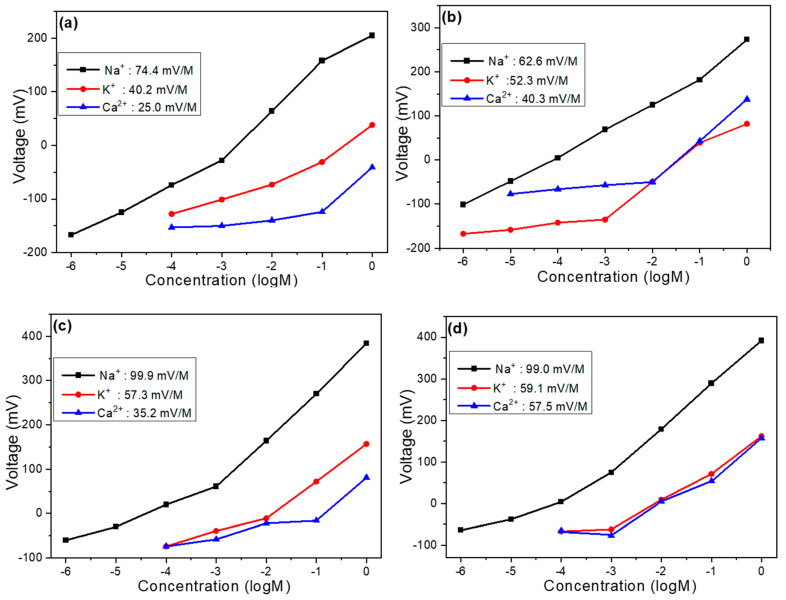
(**a**) Undoped device, (**b**) device with 4 mg GO addition, (**c**) device with 4 mg MWCNT addition, and (**d**) device with 8 mg MWCNT addition: voltage versus concentration relationships for Na^+^, K^+^, and Ca^2+^ ions (selectivity analysis).

Based on our previous analysis, we can confirm that carbon electrodes can effectively serve as reference electrodes for Na^+^ ion components, and the addition of MWCNTs significantly improves the sensing properties of these devices. Moving forward, we investigated how varying amounts of GO or MWCNTs affect selectivity. Figure 6a illustrates the selectivity of the undoped sample toward K^+^ and Ca^2+^ ions. In this experiment, Na^+^ ions exhibited the highest concentration response, followed by K^+^ ions, and then Ca^2+^ ions. Within the concentration range of 1 M to 10^–4^ M, the respective slopes for Na^+^, K^+^, and Ca^2+^ ions were 74.4 mV/dec, 40.2 mV/dec, and 25.0 mV/dec. The highest slope observed for Na^+^ ions indicates superior selectivity of the device toward the target ion. Figure 6b presents selectivity measurements for the three ion types after adding 4 mg of graphene oxide to the film solution. To quantitatively compare selectivity, we used the ratio of response slopes (non-target ion to Na^+^ ion) as a reference metric. For example, within the concentration range of 1 M to 10^–5^ M, the selectivity of the undoped sample toward K^+^ ions is calculated as follows: K^+^ slope/Na^+^ slope = 40.2/74.4 = 0.54. A smaller ratio indicates better selectivity, while a ratio approaching 1 suggests poorer selectivity. After incorporating GO, the selectivity ratio for K^+^ ions increased to 0.83. Similarly, for Ca^2+^ ions, the selectivity ratio rose from 0.34 (undoped) to 0.64 (GO-doped). These results demonstrate that while GO addition may slightly extend the linear range of detection, it simultaneously reduces both the response magnitude toward target ions and overall selectivity. This finding highlights the important trade-off between linear range extension and selectivity that must be carefully considered when optimizing ion-selective electrodes through nanomaterial doping.

Our investigation of selectivity variations in response to different carbon nanomaterial additions continued with the analysis of MWCNT doping effects, as shown in Figure 6c,d, representing samples with 4 mg and 8 mg MWCNT additions, respectively. Measurements were conducted across the concentration range of 1 M to 10^–4^ M to maintain consistent experimental conditions throughout the study. For the sample with 4 mg MWCNT addition, within the specified concentration range, the response slopes for Na^+^, K^+^, and Ca^2+^ ions were measured at 99.9 mV/dec, 57.3 mV/dec, and 35.2 mV/dec, respectively, demonstrating a super-Nernstian behavior for Na^+^ ions that indicates enhanced sensitivity compared to the undoped reference sample. Under these conditions, the calculated selectivity ratios were 0.57 for K^+^ ions and 0.35 for Ca^2+^ ions, values that suggest a slight degradation in selectivity compared to the pristine sample, though this trade-off is considerably less severe than observed with GO doping, and is potentially justifiable given the substantial improvement in sensitivity toward the target Na^+^ ions. The sample with 8 mg MWCNT addition exhibited response slopes of 99.0 mV/dec, 59.1 mV/dec, and 57.5 mV/dec for Na^+^, K^+^, and Ca^2+^ ions, respectively, resulting in selectivity ratios of 0.59 for K^+^ ions and 0.58 for Ca^2+^ ions, indicating further deterioration in selectivity performance, especially pronounced for Ca^2+^ ions, which showed a dramatic reduction in discrimination capability compared to both the undoped sample and the 4 mg MWCNT variant.

This progressive decline in selectivity with increasing MWCNT concentration mainly stems from the creation of additional non-specific binding sites and altered surface charge distribution at higher loading levels, which may facilitate enhanced interaction with divalent Ca^2+^ ions in particular. The comparative analysis across these various doping conditions reveals a critical optimization point: the 4 mg MWCNT addition represents an optimal compromise between enhanced sensitivity (as evidenced by the super-Nernstian response for Na^+^ ions) and maintained reasonable selectivity, particularly when contrasted with the more severely compromised selectivity observed in both the GO-doped sample and the higher-concentration 8 mg MWCNT sample. This finding highlights the importance of precise nanomaterial loading optimization in electrochemical sensor development, where exceeding certain threshold concentrations can lead to diminishing returns in performance, particularly regarding the critical parameter of ion selectivity which ultimately determines the practical utility of the sensor in complex analyte environments containing multiple interfering ionic species. Table 1 presents a comparison of the sensitivity, linearity, and selectivity ratios of carbon electrodes with varying components and concentrations.

**Figure 7:**
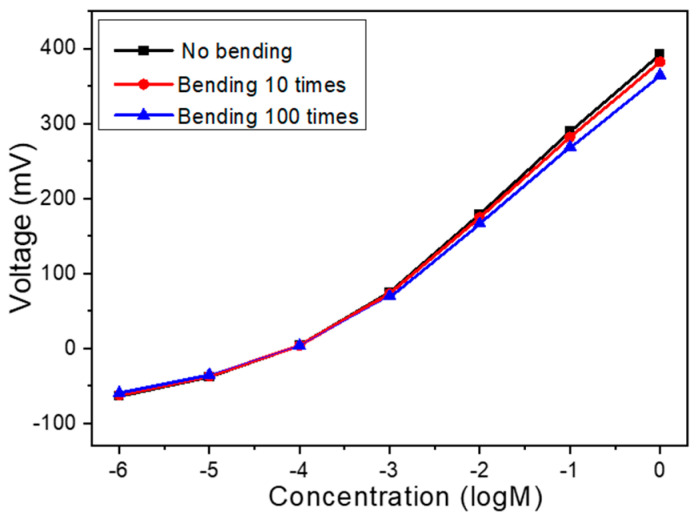
Bending test for the PET-based sensor integrated with 8 mg of MWCNTs.

To evaluate the mechanical durability and morphological stability of the Na^+^ ion sensor under repeated mechanical deformation, bending tests were carried out using the flexible PET-based sensor integrated with 8 mg of MWCNTs in the sensing film. The ion-selective membrane, composed primarily of PVC with tert-butyl calix[4]arene tetraethyl acetate as the Na^+^ carrier and a plasticizer for enhanced film flexibility, was specifically engineered to adapt to flexible substrates. The incorporation of MWCNTs served to reinforce the polymer matrix, forming an interpenetrating network that not only improved electrical conductivity but also significantly enhanced mechanical integrity by distributing stress during deformation. As the results in Figure 7 show, after 10 bending cycles, the sensor maintained approximately 97.5% of its original signal response, and even after 100 cycles, it retained around 94.0%, demonstrating excellent functional stability. Furthermore, post-bending microscopic inspection confirmed that the electrode structure exhibited no visible signs of mechanical damage, such as cracking, delamination, or warping, indicating high morphological stability. This robustness can be attributed to the synergistic effect of the plasticized PVC matrix and the embedded MWCNTs, which collectively endow the film with high flexibility and resilience to repeated mechanical strain. These findings confirm that the sensor is not only functionally reliable under mechanical stress but also structurally stable, thus making it highly suitable for integration into flexible and wearable sensing platforms.

**Figure 8:**
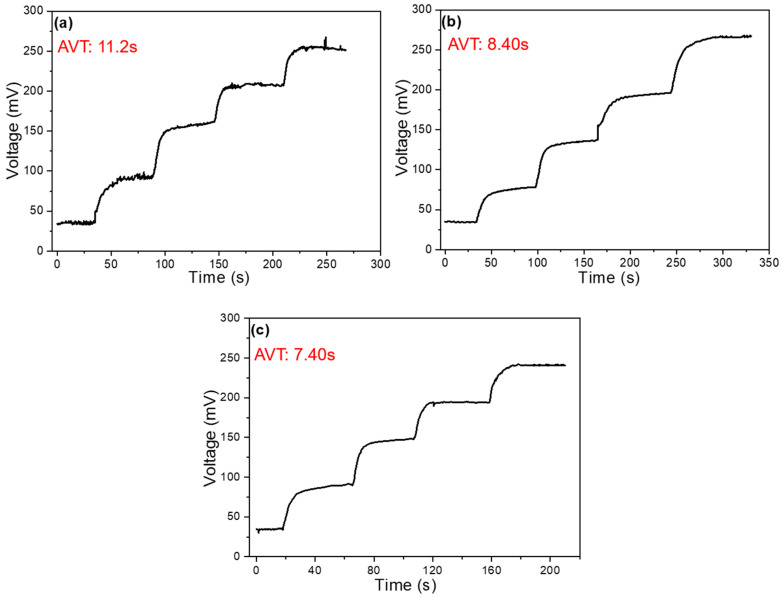
Time-dependent plots of voltage and concentration of a Na^+^ ion sensor (**a**) without the addition of MWCNTs, with the addition of (**b**) 4 mg MWCNTs and (**c**) 8 mg MWCNTs.

The addition of MWCNTs significantly enhances the detection of Na^+^ ions, so we focused on the analysis of response time under conditions with MWCNTs. These data are also an important indicator of the performance of ion-selective electrodes. Figure 8a shows the voltage-time relationship under conditions without the addition of MWCNTs, where the concentration changes over time. NaCl is added at intervals to alter the concentration. When observing the voltage change with respect to concentration over time, we see that the response time decreases as the concentration increases. The concentration changes as follows: 10^−2^ → 3 × 10^−2^ → 10^−1^ → 3 × 10^−1^ → 1 M, and the response time is measured from the moment NaCl is added until the voltage stabilizes. The average response time was calculated to be 11.2 s. Under the conditions with MWCNTs, the response time is faster at higher concentrations. With 4 mg of MWCNTs (as shown in Figure 8b), the average response time is 8.4 s, while with 8 mg of MWCNTs (as shown in Figure 8c), the average response time is 7.4 s. It is clear that the more MWCNTs are added, the faster the response time. Additionally, from Figure 8a, we can observe that the voltage is unstable at different concentrations without MWCNTs, but after the addition of MWCNTs, the voltage becomes stable without any fluctuations. Therefore, it can be concluded that adding MWCNTs improves the stability of the open-circuit voltage measurement.

The improved response time with the addition of MWCNTs can be attributed to the enhanced conductivity and electrochemical properties of the carbon nanotubes. MWCNTs, due to their high surface area and excellent electrical conductivity, facilitate faster ion transfer, which in turn speeds up the system’s ability to reach equilibrium after the concentration change. This is particularly evident in the fact that the response time decreases with higher concentrations of MWCNTs. Furthermore, the increased stability of the voltage after the incorporation of MWCNTs is an important observation. The MWCNTs likely act as a stabilizing agent by reducing the noise and fluctuations in the electrochemical system. This stabilization is crucial for ensuring reliable and accurate measurements in ion-selective electrodes, as fluctuations could lead to erroneous readings and reduced precision. In conclusion, the addition of MWCNTs not only enhances the speed of Na^+^ ions’ detection but also improves the overall stability of the measurement process, making MWCNT-modified electrodes highly promising for practical applications in ion sensing.

As compared in Figure 6 and Figure 8, an increasing concentration of MWCNTs leads to a decrease in sensitivity but a faster response time. The former can be attributed to the creation of additional non-specific binding sites and the altered surface charge distribution at higher loading levels, which may reduce the number of active sites specifically interacting with Na^+^ ions and thus lower the slope of the sensor response. In contrast, the faster response time observed with higher MWCNT content is primarily due to the improved electrical conductivity and electrochemical properties of the carbon nanotubes. The formation of a more efficient conductive network facilitates rapid charge transfer at the sensing interface, thereby accelerating the sensor’s electrical response. Although the presence of additional non-specific binding sites could potentially interfere with ion selectivity and reduce conductivity in localized regions, their overall impact on response time is outweighed by the dominant effect of the enhanced electron transport provided by the well-connected MWCNT network. For Na^+^ ion detection, where rapid signal transduction depends on both ionic diffusion and interfacial electron transfer, the superior electrical pathways formed by MWCNTs significantly reduce charge transfer resistance. As a result, the device exhibits faster response times even in the presence of structural or surface heterogeneities introduced by excess MWCNTs. Therefore, the seemingly contradictory effects can be rationalized by considering that conductivity enhancement plays a more critical role in response speed, while non-specific interactions primarily affect sensitivity.

Due to the wide variety of technologies employed in Na^+^ ion sensors, not every sensor is designed to measure the same set of performance parameters [15,16]. The fundamental sensing mechanisms can differ significantly depending on the materials used, sensor structure, and the type of signal output—some sensors rely on voltage changes, while others measure current responses [17,18]. As a result, the parameters reported across different studies are not always consistent, making it difficult to conduct a comprehensive, one-to-one comparison of overall sensor efficiency. Nevertheless, there are a few critical parameters commonly used to evaluate the performance of Na^+^ ion sensors. These include sensitivity, which refers to the response slope indicating how effectively the sensor detects changes in Na^+^ ion concentration; K K^+^/Na^+^ selectivity, which measures the sensor’s ability to differentiate between sodium and potassium ions; Ca^2+^/Na^+^ selectivity, which assesses how well the sensor discriminates between sodium and calcium ions; and the average response time (Rt), defined as the time required for the sensor’s output voltage to stabilize with each tenfold increase in ion concentration. Moreover, because each sensor may use a different carrier material or membrane composition, and since their response behaviors may vary depending on the specific signal transduction method, it becomes even more challenging to perform a holistic performance comparison across different sensing platforms. Despite these challenges, the Na^+^ ion sensor developed in this study demonstrates strong potential for practical application. It exhibits high selectivity for sodium ions over both potassium and calcium ions, and notably, the best-performing sample achieves a rapid response time of just 7.50 s and has a high sensitivity of 99.0 mV/dec. These values are better than those reported in other research papers [19]. These attributes indicate that the proposed sensor not only performs reliably under competitive ionic conditions but also responds quickly enough for real-time sensing applications.

## 4. Conclusions

When using the carbon electrode as the reference, the voltage ranged from −42 mV to 189 mV, with a sensitivity of 77.0 mV/logM and a linearity of 0.982. For the 10^−4^ M to 1 M range, the K^+^ sensing device with a carbon reference electrode had a small slope over the 10^−4^ M to 1 M range, with voltages shifting from −128 mV to 38 mV and a sensitivity of 41.5 mV/logM. For Ca^2+^ sensing, the voltage response was split into two regions: the voltage changed slightly from −153 mV to −124 mV with a sensitivity of 9.67 mV/logM, while from 10^−1^ M to 1 M, it sharply increased from −124 mV to −41 mV. For samples doped with GO and MWCNTs, the one containing 4 mg of MWCNTs showed the best sensing performance. With 4 mg of MWCNTs, the average response time was 8.4 s, while increasing the MWCNTs to 8 mg reduced the response time to 7.4 s. This demonstrates that a higher MWCNT content leads to faster response times. The response slopes for Na^+^, K^+^, and Ca^2+^ ions were 99.9 mV/dec, 57.3 mV/dec, and 35.2 mV/dec, respectively. Under these conditions, the calculated selectivity ratios were 0.57 for K^+^ ions to Na^+^ ions and 0.35 for Ca^2+^ ions to Na^+^ ions. Notably, the Na^+^ response exhibited super-Nernstian behavior, indicating enhanced sensitivity compared to the undoped reference sample.

## Figures and Tables

**Table 1:** Comparison of the sensitivity, linearity, and selectivity ratio of the carbon electrodes with different components and concentrations.

	Sensitivity	Linearity	Selectivity Ratio
Pure carbon electrode	65.0 mV/dec	0.976	K^+^/Na^+^ = 0.54, Ca^2+^/Na^+^ = 0.34
Carbon electrode with 4 mg GO	60.8 mV/dec	0.992	K^+^/Na^+^ = 0.83, Ca^2+^/Na^+^ = 0.64
Carbon electrode with 8 mg GO	60.8 mV/dec	0.992	K^+^/Na^+^ = 0.84, Ca^2+^/Na^+^ = 0.66
Carbon electrode with 4 mg MWCNTs	74.1 mV/dec	0.937	K^+^/Na^+^ = 0.57, Ca^2+^/Na^+^ = 0.35
Carbon electrode with 8 mg MWCNTs	78.5 mV/dec	0.947	K^+^/Na^+^ = 0.59, Ca^2+^/Na^+^ = 0.58

## Data Availability

The original contributions presented in this study are included in the article. Further inquiries can be directed to the corresponding authors.
